# Alcohol and Road Accidents Involving Pedestrians as Unprotected Road Users

**DOI:** 10.3390/ijerph17238995

**Published:** 2020-12-02

**Authors:** Dorota Lasota, Ahmed Al-Wathinani, Paweł Krajewski, Krzysztof Goniewicz, Witold Pawłowski

**Affiliations:** 1 Department of Experimental and Clinical Pharmacology, Medical University of Warsaw, 02-097 Warsaw, Poland; dorota.lasota@wum.edu.pl; 2 Department of Emergency Medical Services, Prince Sultan Bin Abdulaziz College for Emergency Medical Services, King Saud University, Riyadh 11451, Saudi Arabia; ahmalotaibi@ksu.edu.sa; 3 Department of Forensic Medicine, Medical University of Warsaw, 00-001 Warsaw, Poland; pawel.krajewski@wum.edu.pl; 4 Department of Aviation Security, Military University of Aviation, 08-521 Dęblin, Poland; k.goniewicz@law.mil.pl; 5 Department of Emergency Medicine, Medical University of Lublin, 20-081 Lublin, Poland

**Keywords:** alcohol, ethanol, pedestrians, traffic accidents

## Abstract

According to the World Health Organization (WHO), more than half of all road fatalities involve vulnerable road users, i.e., pedestrians, cyclists, and motorcyclists. Poland is classified as one of the European Union (EU) countries marked by low road safety, with a higher frequency of accidents involving pedestrians compared to other EU countries (31% of all fatalities). Among unprotected road users, a significant group of victims are pedestrians, who are often under the influence of alcohol. This study aims to analyze the impact of alcohol on the risk of occurrence and consequences of road accidents among pedestrians. The source of data was the medical documentation of the Department of Forensic Medicine of the Medical University of Warsaw. In more than half of pedestrian deaths, the presence of alcohol was found; regardless of the place of the event and the place of death, among the victims under the influence of alcohol, males dominated; the average age of the victims under the influence of alcohol was significantly lower compared to the average age of sober victims, with younger victims being significantly more likely to die at the scene of the accident, especially in rural areas; significantly higher alcohol concentrations were found in males, in victims who died at the scene of the accident, and with victims of accidents in rural areas. Among pedestrian traffic accident fatalities, the most numerous group comprised young men under the influence of alcohol. In rural areas, a higher percentage of pedestrian victims died at the scene as a result of excessive alcohol consumption. These areas should be subject to intensive preventive measures to increase the safety of pedestrians as unprotected road users.

## 1. Introduction

Unprotected road users constitute 46% of the global percentage of road fatalities [1]. According to Polish Police statistics for the years 2009–2019, the lowest percentage of unprotected road traffic participants among all victims of road accidents was recorded in 2009, i.e., 37.0% [2]. In 2014, it was as much as 43.5%, while currently unprotected road users constitute 40.0% of all road accident victims [3,4,5,6,7,8,9]. This group, therefore, deserves special attention, because unlike road users using cars, they are not protected by the car body and cannot rely on airbags or seat belts. Statistics of road accidents in Poland show that a significant group of participants in road accidents are pedestrians. During the decade (2009–2019), 104,036 accidents were recorded (26.5% of total road accidents), in which 11,842 pedestrians were killed (31.7% of the total), (Table 1).

The main causes of accidents involving these pedestrians under the influence of alcohol are careless stepping onto the road in front of a moving vehicle, lying down, sitting, standing, or even sleeping on the road [2]. Most accidents and casualties among pedestrians are caused by vehicle drivers, especially drivers of passenger cars [10,11,12]. Such accidents are mostly “running over a pedestrian" [13]. Most pedestrian accidents occur in built-up areas, but the consequences of accidents occurring outside built-up areas are more tragic and often result in death at the scene of the accident. The reasons for this can be attributed, among other things, to poorly lit roads outside built-up areas, which make pedestrians less visible, especially during bad weather conditions [14,15]. Furthermore, excessive speeding of vehicles outside built-up areas, the later arrival of specialist assistance at the scene of the accident, and the state of intoxication of pedestrian road users also contribute to this [2].

This study aims to analyze the impact of alcohol on the risk of occurrence and the consequences of traffic accidents among pedestrians as unprotected road users.

## 2. Materials and Methods

### 2.1. Materials

We obtained data from the documentation of the Department of Forensic Medicine (DFM) of the Medical University of Warsaw. Retrospective analysis included 321 out of 370 pedestrian traffic accident victims from the Warsaw area, recorded in the DFM death records. The analysis took into account the gender and age of the victims, mechanism of injury, bodily injuries, place of death (at the scene, at the hospital), place of event, i.e., the area of the Warsaw metropolis (Warsaw city, urban area, and rural areas). Although customarily in research, the scene of the incident is divided into built-up and undeveloped areas, based on the available materials, it would be difficult to unequivocally assess whether we are dealing with a built-up area or not, because according to the Polish codex definition, the beginning and end of the road section running through a built-up area are marked with appropriate information signs, which is practically impossible to verify based on documentation alone. Therefore, the proposed tripartite division seems to be more precise. In official statistics, urban and rural areas are distinguished based on the territorial division of the country. Urban areas (cities) include areas located within the administrative boundaries of cities, i.e., areas of urban communes and cities in urban–rural communes. Rural areas (villages) include areas outside the administrative borders of cities, which include areas of rural communes and rural parts of urban–rural communes. Warsaw is the largest city in Poland in terms of population and area. The analysis also included ethyl alcohol concentration in blood, muscles, and vitreous humor, determined based on toxicological examination performed by gas chromatography, expressed as per mille (‰). The obtained data were analyzed by comparing two groups, i.e., pedestrian victims under the impact of ethyl alcohol at the time of the accident (≥0.2‰), *n* = 166, and sober pedestrian victims (0.0‰), *n* = 155. We eliminated unexplained deaths from this analysis, as to the place of the event, place of death, and type of vehicle involved in the event, *n* = 49.

### 2.2. Methods

Statistical analyses were carried out using IBM SPSS Statistics version 24. A series of chi-square analyses, Pearson correlation analyses, a series of one-factor ANOVA variance analyses, Student’s t-analyses, and logistic regression analysis were performed. The typical threshold of *p* < 0.05 was assumed as the level of significance; however, the results of the probability of test statistics at the level of 0.05 < *p* < 0.1 were interpreted as significant at the statistical trend level.

## 3. Results

In the analyzed population, *n* = 214 were men (66.67%). The age of the examined victims was varied, ranging from three to ninety-one years, with the average age being 54 ± 20.34 years. Alcohol was found in 166 of the victims (51.71%). The largest part (*n* = 283) of the investigated pedestrians who were struck experienced injuries to numerous regions of the body (88.16%), death occurred at the crash site for 217 of the victims (67.60%), and *n* = 212 victims were injured in crashes due to passenger cars (66.04%). The results are presented in Table 2.

Regardless of where the accident occurred, men dominated the group under the influence of alcohol. In the group of sober victims, a slight predominance of women was observed. The size of the effect was high for rural areas, slightly lower for Warsaw, and moderate for urban areas of the Warsaw area (Table 3).

Additionally, regardless of where the accident occurred, the average age of the victims under the influence of alcohol was significantly lower compared to the average age of sober victims. The strongest effect of this group was observed in Warsaw (accordingly: 45.96 ± 16.11 vs. 65.61 ± 20.41 years, t = −5.71 *p* < 0.001, *d* = 1.03).

Chi-squared analysis, taking into account the group and place of the accident, divided into two age categories, i.e., younger and older victims of accidents (*Me* = 55), showed that younger victims affected by alcohol dominated in rural areas of Warsaw, while older victims were sober in Warsaw. In both cases, the size of the effect was moderate (Table 4).

When considering group and gender by location of death, it was found that in the group under the influence of alcohol, the majority were men, both when the death occurred at the scene of the accident and within the first day of hospitalization. In the group of sober victims, a slight predominance of women was observed, especially when death occurred at the scene of the accident. When death occurred within the first day of hospitalization, the groups were the same. The size of the effect was quite high in both cases (Table 5).

Irrespective of the location of death, the average age of victims under the influence of alcohol was significantly lower compared to the average age of sober victims. The size of the effect of the group for both variants was equally high (Table 6).

The analysis taking into account the group and location of death, split into two age categories, showed that younger victims under the influence of alcohol died more often at the scene of the accident, while sober older victims died on the first day of hospitalization. The size of the effect was moderate but slightly weaker than that in the group of younger victims, *Vc* = 0.23 (Table 7).

Frequency analysis was also performed by location of accident, location of death, and group. Differences were found at the border of the statistical trend in the victims under the influence of alcohol, in which the highest number was recorded in rural areas of Warsaw, especially those who died at the scene of the accident, *p* = 0.058. The size of the effect was moderate, *Vc* = 0.19 (Table 8).

The presence of ethyl alcohol was noticed in more than 51% of fatal pedestrian victims. Its concentration was 2.05 ± 0.895 per mille and it was in the range of 0.2–4.4‰, with ethanol concentration in men being statistically higher (2.12 ± 0.87) compared to the women’s concentration (1.69 ± 0.95, *p* = 0.021, *d* = 0.49).

An analysis of Pearson’s *r* correlation between alcohol level and age was performed. The relationship proved to be statistically insignificant, which means that there was no correlation between age and alcohol level, *r* = 0.05, *p* = 0.487.

Analogous correlation analysis was performed with additional consideration of the gender of the victims. The analysis did not show statistically significant differences, which means that no correlation was observed between age and alcohol level in either women or men (respectively: *r* = 0.03, *p* = 0.686 vs. *r* = 0.15, *p* = 0.463).

Analysis of the alcohol level by type of injury showed differences at the border of the statistical trend. The alcohol level was found to be lower in victims who suffered injuries to numerous regions of the body, compared to those who suffered other bodily injuries (respectively, 2.01 ± 0.89 vs. 2.38 ± 0.91 per mille, *F* = 3.02, *p* = 0.084).

One-way analysis of variance, taking into account alcohol levels and the location of the accident, showed the highest concentration of alcohol in accident victims in rural areas of the Warsaw area (2.22 ± 0.81 per mille, *F* = 5.36, *p =* 0.006).

Analysis of the alcohol concentration by location of death showed differences at the borderline of the statistical trend, which means that victims who died at the scene of the accident had a higher alcohol concentration than those who died on the first day of hospitalization (respectively 2.11 ± 0.90 vs. 1.77 ± 0.83 per mille, *F* = 3.56, *p* = 0.061).

One-way analysis of the variance including alcohol concentration and the injury mechanism showed no statistically significant differences, which means that the alcohol concentration of pedestrian victims did not differ according to the type of vehicle involved in the event, *F* = 0.31, *p* = 0.87.

A logistic regression analysis was also conducted to examine the simultaneous impact of all measured predictors on the probability of death in a road accident among alcohol-drunk pedestrians. The applied regression model (Nagelkerke R^2^) proved to be well suited to the data Χ^2^ (9) = 129.73; *p* < 0.001. The fit of the model was also confirmed by the insignificant result of the Hosmer and Lemeshow test Χ^2^ (8) = 6.83; *p* = 0.555. The constructed model explains 44.3% of the variance, obtaining the classification correctness at the level of 76.9%. Most of the predictors turned out to be statistically significant. The analysis showed that the chance of death in a road accident among pedestrians under the influence of alcohol was more than five times higher in men compared to that in women and decreased with age by about 4% with each year of life. Moreover, the obtained results showed that pedestrians under the influence of alcohol were more likely to die in incidents involving passenger cars. The type of sustained injuries turned out to be insignificant, but the place of the accident and the place of death turned out to be significant variables. Thus, the chance of death in a road accident among pedestrians under the influence of alcohol was about twice as high in urban and rural areas of the Warsaw agglomeration compared to that in Warsaw Capital City, and the chance of death on the first day of hospitalization compared to that of death at the scene of the accident was approximately three times smaller (Table 9).

## 4. Discussion

Although the risk of injury and death impacts all road users, there are significant differences in mortality rates between groups of victims. Pedestrians are a high-risk group [1,5].

According to annual reports from the Polish Police for the years 2011–2019, the greatest number of accidents in which pedestrians died were caused by drivers of passenger cars (for the years 2009 and 2010, there were no such data). The highest number of such events was recorded in 2011 (5486 accidents, from which 423 were killed) and the lowest in 2019 (3769, from which 257 were killed) [9]. Similarly, in the presented study, the majority of accidents in which pedestrians were killed were caused by passenger car drivers (Table 2 and Table 9). In the group of sober victims, this was also the leading mechanism of injury, although there were also nearly twice as many victims of accidents involving lorries, vans, and buses, and about five times as many victims of tram accidents. Taking into account the fact that nearly 75% of sober victims died in the urban areas of the Warsaw area, with as much as 60.00% of them in Warsaw, it seems that the mechanism of injury is determined by the location of the accident.

According to WHO data, 75% of all people who die in road accidents worldwide are male [16]. In the presented analysis, men constituted nearly 67% of the studied population. It is believed that such a gender distribution among victims is probably caused by an increased tendency to take risky actions in the male group, especially under the influence of alcohol [5]. In the presented analysis, the gender imbalance was particularly significant in the group of pedestrians under the influence of alcohol, where there were more than five times more men than women. According to the Central Statistical Office (GUS), in 2014, the actual mortality rate per 100,000 population due to road accidents in Poland was 8.4 [17]. However, the risk of life for men is many times higher than that for women (standardized coefficient of road accident mortality for men is 12.4 vs. 3.3 for women). The phenomenon of high mortality due to road accidents in rural areas, for which the standardized death rates due to road accidents were 16.9 for men and 4.2 for women, is also characteristic. In 2016, these rates were 17.8 for men and 4.6 for women [3,4]. The results obtained in this study are in line with this trend.

It is customary for studies presented in the literature to separate the location of the accident into built-up and non-built-up areas [18,19,20]. This is undoubtedly important in the context of the speed reached by vehicles, which has an impact on the consequences of traffic accidents. "At high vehicle speeds, pedestrians and cyclists suffer the most severe consequences of accidents. It is in these cases when the kinetic energy released during an impact must be counteracted by the resilience of the human body. The results of the study indicate that when a vehicle impacts at 30 km/h, 9 out of 10 pedestrians will survive the accident; at 50 km/h, half of them will die; and at a speed exceeding 60 km/h, 9 out of 10 will die. These values are higher for older pedestrians (>60 years)" [21].

It is worth mentioning at this point that in Poland the speed limits are set following the rule of law, and appropriately to whether or not it is a built-up area. However, according to the law, there may be local restrictions other than the standard ones for built-up areas (e.g., changing the speed limit in a built-up area from 50 km/h to a maximum of 70 km/h in places where there are pedestrian crossings) [21]. Furthermore, the actual speed of the vehicle involved in an accident is normally the subject of an investigation into the circumstances of the accident, the findings of which remain classified. Thus, as the speed of the vehicle increases, the kinetic energy released during a collision also exponentially increases, increasing the risk of death and serious injury. Regarding the available materials, it would be difficult to assess whether or not we are dealing with a built-up area, because according to the Polish definition of the code, the beginning and the end of a section of road passing through a built-up area are marked by appropriate road signs, which is practically impossible to verify based on documentation alone [22]. This study also did not examine the cause–effect relationships, so the three divisions into the Capital City of Warsaw and rural and urban areas of the Warsaw area seemed to be more precise. It is also a source of new, valuable information in the context of the epidemiology of traffic accidents involving pedestrians.

The consequences of this study established that victims were mainly younger men (*Me* = 55) under the influence of alcohol, and the highest rates of these types of incidents were observed in rural areas. These victims were most often killed at the scene of the accident. According to the study of de Carvalho Ponce et al. on the impact of alcohol in traffic crashes in Sao Paulo, the victims of road crashes were mostly men aged 25–34 years. Similar consequences have been mentioned by Gjerde et al., who analyzed the effect of alcohol and psychoactive substances on road crashes in Norway, as well as Hickox et al., who analyzed pedestrian mortality in Clark County, Nevada. De Boni et al., in their research on factors related to alcohol consumption and drugs by participants of road crashes in southern Brazil, demonstrated that the victims were also mainly men, with the average age being 37 years [23,24,25,26].

Studies on the role of the impact of alcohol in road accidents indicate that ethyl alcohol is a factor that significantly impacts their occurrence and consequences. This study showed that pedestrians under the influence of alcohol are significantly more likely to die at the scene of the accident, and the frequency of this rises with the increase in alcohol concentration. The increase was especially significant in the rural parts of the Warsaw area, where the substantial majority of the pedestrians also died at the scene of the road crash. However, this does not necessarily have to be the result of injuries, although indeed almost all pedestrians were found to have received injuries in numerous regions of the body, i.e., multiple injuries representing injuries to at least two or more regions of the body, each of which is an indication for hospital treatment.

It seems that the location of the accident itself may be of significance here. The most numerous group of pedestrian deaths was recorded in Warsaw city (43.30%). This result is not surprising, considering the specificity of Warsaw as a place with an exceptionally high concentration of traffic. According to reports on the State of Road Traffic Safety in the Capital City of Warsaw for the years 2009–2019, the most common type of accidents on the capital’s streets were those involving pedestrians. The highest number of such events, i.e., 492 (45.8% of the total) and 38 killed (66.6% of the total), occurred in 2010, while the lowest number occurred in 2019, i.e., 302 (33.4% of the total) and 21 killed (60.0% of the total). Although their number is decreasing, the pedestrian is still the most vulnerable road user on the capital’s streets [23,24,25]. The percentage of pedestrian deaths in rural areas was slightly lower (38.94%), but the percentage of victims under the influence of alcohol was the highest (51.81%). Sober pedestrians accounted for only 25.16%. The lowest percentage of pedestrian victims was in urban areas of the Warsaw area (17.76%), where sober pedestrians constituted 14.84%, while in the Capital City of Warsaw it was as much as 60%.

In contrast to pedestrians under the influence of ethyl alcohol, who constitute nearly 82% of cases who died at the scene of the accident, sober pedestrians died on the first day of hospitalization in over 47% of cases. Similar conclusions are presented in the Road Safety Report in Poland, according to which the most numerous and most severely impacted group of victims compared to other road users are pedestrians, who statistically more often die at the scene of the accident or during hospitalization and represent a higher percentage of the severely injured [27,28].

According to legal standards in Poland, when the blood alcohol content is or nears a concentration of 0.2‰ to 0.5‰ (or the presence of 0.1 mg to 0.25 mg of alcohol in 1 dm^3^ in exhaled air), this is considered a state after alcohol consumption [22]. When the blood alcohol level is or nears a blood alcohol level above 0.5‰ (and there is more than 0.25 mg in 1 dm^3^ in the exhaled air), we are talking about a state of intoxication [29,30,31,32]. Unfortunately, in the presented analysis, the above-mentioned limits were significantly exceeded in pedestrians (0.2–4.40‰). A comparison of ethyl alcohol concentration in pedestrian victims showed that alcohol consumption in pedestrian victims was significantly higher in men than in women. The highest alcohol concentrations were recorded in the victims who died at the scene of the accident, especially in rural areas of the Warsaw area.

## 5. Limitations

The presented analysis has some limitations, as it only takes into account the fatalities of pedestrian traffic accidents. This study also did not examine the cause–effect relationships, and it also does not take into account factors other than the presented risks affecting pedestrian safety, such as, for example, poorly lit roads, traffic intensity, season of the year, bad weather conditions, or the speed at which vehicles are moving. Furthermore, the Department of Forensic Medicine, from which we drew our data, is the major referral center in Warsaw and the only unit of this type at the Medical University of Warsaw. According to the importance of the problem, it is necessary to conduct further in-depth research in this area.

## 6. Conclusions

Alcohol is an important risk factor for road accidents involving pedestrians as unprotected road users. The most numerous group of road accident fatalities among pedestrians are men, with a particular emphasis on men under the influence of alcohol. In particular, victims under the influence of alcohol were more likely to die in crashes, when the place of the accident was in a rural area [33]. In these circumstances, death at the scene of an accident is equally common. Alcohol is of course one of the many factors that impact their safety; undoubtedly other factors include badly lit roads (outside built-up areas, roads are not illuminated, so pedestrians are less visible), the level of traffic intensity, the time of year (especially in autumn and winter, when visibility is worse in the earlier twilight), bad weather conditions, or the aforementioned speed at which vehicles are moving [34]. Death at the scene of the crash might be caused, among other things, by the inferior quality of pre-hospital care. Delays in detecting and providing care for those involved in a road traffic crash increase the severity of injuries. Intensive prevention programs ought to be implemented in these areas so that the safety of pedestrians can be improved [20,35,36,37,38,39]. This requires involvement from multiple sectors, such as police, health, education, transport, and actions that address the safety of roads, vehicles, and road users [40].

## Figures and Tables

**Table 1:** Road accidents involving pedestrians in Poland, 2009–2019.

Year	Road Accidents	Deaths	Accidents Involving Pedestrians	% of Total	Deaths	% of Total
**2009**	44,196	4572	12,834	29.0	1477	32.3
**2010**	38,832	3907	11,286	29.0	1245	31.9
**2011**	40,065	4189	11,220	27.9	1419	33.9
**2012**	37,046	3571	10,309	27.8	1167	32.7
**2013**	35,847	3357	9489	26.5	1147	34.2
**2014**	34,970	3202	9106	26.0	1127	35.2
**2015**	32,967	2938	8581	26.0	923	31.4
**2016**	33,664	3026	8461	25.1	868	38.7
**2017**	32,760	2831	8197	25.0	873	30.8
**2018**	31,674	2862	7548	23.8	803	28.1
**2019**	30,288	2909	7005	23.1	793	27.3

Source: Own elaboration based on statistics, road accidents—annual reports.

**Table 2:** Distribution of analyzed variables.

Variables	Alcohol Group *n* = 166 (51.71%)	No Alcohol Group *n* = 155 (48.29%)	*p* Value
**Gender *n (%)***			
Women	27 (16.27)	80 (51.61)	*p* < 0.001
Men	139 (83.73)	75 (48.39)	
**Age *n (%)***			
Mean	45.87 ± 14.20	62.72 ± 22.26	*p* < 0.001
Median	48	71	
Minimum	15	3	
Maximum	86	91	
**Bodily injuries *n*%**			
Numerous regions of the body *	146 (87.95)	137 (88.39)	*p >* 0.05
Other	20 (12.05)	18 (11.61)	
**Location of accident *n (%)***			
Warsaw city	46 (27.71)	93 (60.00)	*p* < 0.001
Urban areas	34 (20.48)	23 (14.84)	
Rural areas	86 (51.81)	39 (25.16)	
**Death place *n (%)***			
At the scene of the accident	136 (81.93)	81 (52.26)	*p* < 0.001
Within 1 day of hospitalization	30 (18.07)	74 (47.74)	
**Mechanism of injury *n (%)***			
Passenger car	121 (72.89)	91 (58.71)	*p <* 0.05
Lorry/delivery truck/bus	21 (12.65)	32 (20.64)	
Tram	4 (2.41)	17 (10.97)	
Train	16 (9.64)	11 (7.10)	
Other vehicle	4 (2.41)	4 (2.58)	

* Multiple injuries.

**Table 3:** Number of victims by group, gender, and location of accident.

Location of Accident			Under the Influence of Alcohol	Sober	Χ^2^	Significance	φ
Warsaw city	Women	Number	7	47	16.16	<0.001	0.34
		% of the group	15.22%	50.54%			
	Men	Number	39	46			
		% of the group	84.78%	49.46%			
Urban areas	Women	Number	8	12	4.94	0.026	0.29
		% of the group	23.53%	52.17%			
	Men	Number	26	11			
		% of the group	76.47%	47.83%			
Rural areas	Women	Number	12	21	21.98	<0.001	0.42
		% of the group	13.95%	53.85%			
	Men	Number	74	18			
		% of the group	86.05%	46.15%			

Χ^2^—the result of the chi-square test.

**Table 4:** Number of victims by group, location of accident, and age category.

			Under the Influence of Alcohol	Sober	Χ^2^	Significance	Cramér’s *V*
Younger	Warsaw city	Number	32	23	9.86	0.007	0.24
		% of the group	26.02%	51.11%			
	Urban areas	Number	22	7			
		% of the group	17.89%	15.56%			
	Rural areas	Number	69	15			
		% of the group	56.10%	33.33%			
Older	Warsaw city	Number	14	70	12.08	0.002	0.28
		% of the group	32.56%	63.64%			
	Urban areas	Number	12	16			
		% of the group	27.91%	14.55%			
	Rural areas	Number	17	24			
		% of the group	39.53%	21.82%			

Χ^2^—the result of the chi-square test.

**Table 5:** Number of victims by group, gender, and location of death.

			Under the Influence of Alcohol	Sober	Χ^2^	Significance	φ
At the scene of the accident	Women	Number	23	43	31.39	<0.001	0.38
		% of the group	16.91%	53.09%			
	Men	Number	113	38			
		% of the group	83.09%	46.91%			
Death within 1 day of hospitalization	Women	Number	4	37	12.02	0.001	0.34
		% of the group	13.33%	50.00%			
	Men	Number	26	37			
		% of the group	86.67%	50.00%			

Χ^2^—the result of the chi-square test.

**Table 6:** Average age of victims by group and location of death.

	Under the Influence of Alcohol		Sober				95% CI		
	M	SD	M	SD	*t*	*p*	LL	UL	Cohen’s *d*
At the scene of the accident	45.16	14.10	59.93	21.89	−5.44	<0.001	−20.14	−9.39	0.85
Death within 1 day of hospitalization	49.07	14.47	65.78	22.41	−4.51	<0.001	−24.10	−9.33	0.82

M—average (for age), SD—standard deviation, *t*—Student’s *t*-test result, *p*—significance level, 95% CI—confidence interval, LL, UL—is the lower and upper limits of the confidence interval.

**Table 7:** Number of victims by group, location of death, and age category.

			Under the Influence of Alcohol	Sober	Χ^2^	Significance	Cramér’s *V*
Younger	At the scene of the accident	Number	105	29	8.93	0.003	0.23
		% of the group	85.37%	64.44%			
	Death within 1 day of hospitalization	Number	18	16			
		% of the group	14.63%	35.56%			
Older	At the scene of the accident	Number	31	52	7.67	0.006	0.22
		% of the group	72.09%	47.27%			
	Death within 1 day of hospitalization	Number	12	58			
		% of the group	27.91%	52.73%			

Χ^2^—the result of the chi-square test.

**Table 8:** Number of victims by location of accident, location of death, and group.

			Warsaw city	Urban Areas	Rural Areas	Χ^2^	Significance	Cramér’s *V*
Under the influence of alcohol	At the scene of the accident	Number	33	27	76	5.78	0.058	0.19
		% of location of accident	71.74%	79.41%	88.37%			
	Death within 1 day of hospitalization	Number	13	7	10			
		% of location of accident	28.26%	20.59%	11.63%			
Sober	At the scene of the accident	Number	43	13	25	3.71	0.152	0.16
		% of location of accident	46.24%	56.52%	64.10%			
	Death within 1 day of hospitalization	Number	50	10	14			
		% of location of accident	53.76%	43.48%	35.90%			

Χ^2^—the result of the chi-square test.

**Table 9:** Results of the logistic regression analysis testing the predictors of death in a road accident among pedestrians under the influence of alcohol.

	B	SE	Χ^2^	*p*	OR	OR 95% CI	
						LL	UL
Gender *	1.63	0.32	26.48	<0.001	5.08	2.73	9.43
Age	−0.04	0.01	20.43	<0.001	0.97	0.95	0.98
Passenger car			8.71	0.069			
Lorry/delivery truck/bus	−0.80	0.38	4.44	0.035	0.45	0.21	0.95
Tram	−1.27	0.71	3.22	0.073	0.28	0.07	1.13
Train	−0.94	0.51	3.46	0.063	0.39	0.14	1.05
Other vehicle	−0.55	0.86	0.41	0.520	0.58	0.11	3.09
Bodily injuries **	0.01	0.45	0.00	0.980	1.01	0.42	2.44
Warsaw city			9.46	0.009			
Urban areas	0.87	0.40	4.68	0.031	2.39	1.09	5.24
Rural areas	0.94	0.33	8.35	0.004	2.57	1.36	4.87
Death place ***	−1.19	0.32	13.98	<0.001	0.30	0.16	0.57

* Men compared to women, ** other types of injuries compared to injuries to numerous regions of the body, *** death within 1 day of hospitalization compared to death at the scene of the accident. B—regression coefficient, SE—standard error, Χ^2^—the result of the chi-square test, OR - odds ratio, OR 95% CI—confidence interval, LL, UL—lower and upper limits of the confidence interval.
